# Structural and Magnetic Characterization of Mechanically Alloyed (Fe_2_O_3_)_1−x_(Al_2_O_3_)_x_ Solid Solutions via Pulsed Neutron Powder Diffraction

**DOI:** 10.3390/ma18091911

**Published:** 2025-04-23

**Authors:** Dong Luo, Hayato Nakaishi, Takeshi Yabutsuka, Takashi Saito, Takashi Kamiyama, Masato Hagihala, Shigeomi Takai

**Affiliations:** 1 Graduate School of Energy Science, Kyoto University, Kyoto 606-8501, Japan; luo.dong.56s@st.kyoto-u.ac.jp (D.L.); yabutsuka@energy.kyoto-u.ac.jp (T.Y.); 2 High Energy Accelerator Research Organization (KEK), Ibaraki 305-0801, Japan; saitot@post.kek.jp (T.S.); takashi.kamiyama@kek.jp (T.K.); hagihara.masato@jaea.go.jp (M.H.)

**Keywords:** neutron diffraction, mechanical alloying, corundum-type structure, solid solution, magnetic moment, anode material

## Abstract

Neutron powder diffraction experiments were carried out to characterize mechanochemically synthesized (Fe_2_O_3_)_1−x_(Al_2_O_3_)_x_ solid solutions with corundum-type structure, focusing on their lattice and magnetic structures with varying temperature and composition. The neutron diffraction experiments for (Fe_2_O_3_)_0.5_(Al_2_O_3_)_0.5_ in the temperature range between 4 K and 300 K reveal that no significant structural phase transition occurred. The behavior of temperature variation of lattice parameters is different from α-Fe_2_O_3_ and α-Al_2_O_3_ and reveals the thermal expansion coefficients of α*_a_* = 5.76(2) × 10^−6^ K^−1^ and α*_c_* = 6.19(5) × 10^−6^ K^−1^ between 200 K and 300 K. The room temperature neutron diffraction of (Fe_2_O_3_)_1−x_(Al_2_O_3_)_x_ shows a linear decrease in lattice parameters with the aluminum substitution, following Vegard’s law, along with a decrease in the magnetic moment, indicating the dilution effect on spin interactions. With the increase in the aluminum substitution from x = 0 to 0.5, the deduced magnetic moment decreases from 2.224 *μ*_B_ to 0.862 *μ*_B_.

## 1. Introduction

Corundum-type structured α-Fe_2_O_3_ (hematite) is a promising candidate anode material for lithium-ion batteries (LIBs), though significant volume changes during its conversion reaction restrict its cycle performance [1,2,3,4,5]. Using α-Al_2_O_3_ (corundum) to stabilize the crystal structure of α-Fe_2_O_3_ by forming a solid solution seems an appropriate means of addressing this limitation. However, the solid solution ranges between them in the equilibrium phase diagram are considerably limited around the end-number compositions [6,7]. Despite extensive efforts to extend the solid solution range, the wide range of the intermediate composition (0.3 < x < 0.75) for (Fe_2_O_3_)_1−x_(Al_2_O_3_)_x_ exhibits two-phase coexistence [8,9,10,11]. In recent years, we have innovatively synthesized the entire compositional range (0 ≤ x ≤ 1) of the corundum-type solid solution through the mechanical alloying method starting from a mixture of γ-Fe_2_O_3_ and γ-Al_2_O_3_ [12]. X-ray diffraction confirmed that the compositional dependence of lattice parameters of the obtained solid solutions agrees with Vegard’s law assuming JCPDS data of α-Fe_2_O_3_ (33-664) and α-Al_2_O_3_ (46-1212) [12,13]. In the charge–discharge tests of (Fe_2_O_3_)_1−x_(Al_2_O_3_)_x_ as electrode materials, the solid solutions exhibited good cycle performance in the compositional range of 0.33 ≤ x ≤ 0.45 [12].

In previous work, we carried out transmission electron microscopy (TEM), extended X-ray absorption fine structure (EXAFS), and Mössbauer spectroscopy experiments to clarify the atomic level solid solution formation and the cation distribution in samples with x = 0 − 0.67 [14]. The TEM experiments showed that the typical particle size of the solid solutions was 10–20 nm, with lattice spacings consistent with those calculated with Vegard’s law. The EXAFS measurements showed that the radial distribution of Fe-Fe distance decreased monotonously with the increase in aluminum substitution, while the Fe-O distance did not significantly. Through room-temperature Mössbauer spectroscopy, with the increase in the aluminum substitution, the spectra gradually shifted from a sextet pattern (weak ferromagnetism) into a doublet (paramagnetism) due to the dilution of the Fe-Fe spin interaction. To further understand the thermodynamic behavior of the (Fe_2_O_3_)_1−x_(Al_2_O_3_)_x_ solid solutions, low-temperature heat capacities have been measured [15]. While the obtained heat capacities of the solid solutions showed a similar trend of temperature dependence with pure α-Fe_2_O_3_ and α-Al_2_O_3_ [16], the measured values are slightly higher than their weighted averages, and a heat capacity anomaly was observed below 25 K.

To investigate the detailed structural information of the mechanically alloyed (Fe_2_O_3_)_1−x_(Al_2_O_3_)_x_ solid solution, we conducted time-of-flight (TOF) neutron powder diffraction experiments in the present study. Neutron diffraction provides precise atomic positions including oxygen ions [17,18] and also probes the magnetic structure due to the neutron spin interactions, which matches the investigation of a new type of solid solution with spin interactions [19]. In this study, we employed the neutron powder diffraction technique to clarify the structural and magnetic properties of the (Fe_2_O_3_)_1−x_(Al_2_O_3_)_x_ solid solution with varying temperature and composition.

## 2. Materials and Methods

(Fe_2_O_3_)_1−x_(Al_2_O_3_)_x_ solid solutions were synthesized through high-energy ball milling, as described in the previous studies [12,14,15]. Stoichiometric mixtures of γ-Fe_2_O_3_ (Sigma Aldrich, St. Louis, MO, USA, 99+%) and γ-Al_2_O_3_ (Strem Chem., Newburyport, MA, USA, 96%) powders were loaded into a 20 mL volume silicon nitride (Si_3_N_4_) milling vessel, along with ten silicon nitride milling balls in 10 mm diameter. No solvents were used during the process. The milling was conducted in air under atmospheric pressure using a planetary ball milling machine (Fritsch, Idar-Oberstein, Germany, Premium Line P-7) operated at 800 rpm in segmented intervals for a total milling time of 240 min (12 cycles of 20 min of operation with 40 min of cooling).

The neutron powder diffraction experiments were carried out using the special environment neutron diffraction instrument (BL09 SPICA) at the Japan Proton Accelerator Research Complex (J-PARC) in Tokai, Japan [20]. The instrument features a top-loading 4 K cryostat with a measurement range from 4 K to 300 K. About 1 g of synthesized (Fe_2_O_3_)_1−x_(Al_2_O_3_)_x_ solid solution powder with x = 0.5 was loaded into a vanadium sample holder, which was sealed under helium gas conditions. The diffraction data were collected at room temperature for 3 h, 200 K for 2.5 h, 100 K for 2.5 h, 20 K for 4.5 h, and 4 K for 4.5 h through the BS (Back Scattering) bank for *d* = 0.3–4.0 Å and QA (Quasi Axial) bank for *d* = 0.4–5.0 Å. BS and QA detectors are positioned at different scattering angles, with BS located at high angles for high-resolution structural analysis and QA at intermediate angles offering balanced resolution and intensity, making it suitable for detecting magnetic scattering. Compositional dependence was also investigated using x = 0, 0.20, 0.40, and 0.50. We selected these compositions based on the Mössbauer spectroscopy results [14], which show a transition from a sextet to a doublet within this range. The diffracted data were collected at room temperature for 3 h. Lattice information was mainly obtained from the BS bank data, while the magnetic information was from the QA bank. The lattice and magnetic structures were refined by the Rietveld method using Z-Rietveld software (ver. 2.0.0) [21]. For the crystal structure analysis of the solid solutions, the space group was selected as *R*$\bar{3}$*c* of hematite lattice, placing Fe and Al at the identical 12*c* and O at the 16*e* sites.

## 3. Results and Discussion

### 3.1. Temperature Dependences of the Structure of (Fe_2_O_3_)_0.5_(Al_2_O_3_)_0.5_

**Figure 1:**
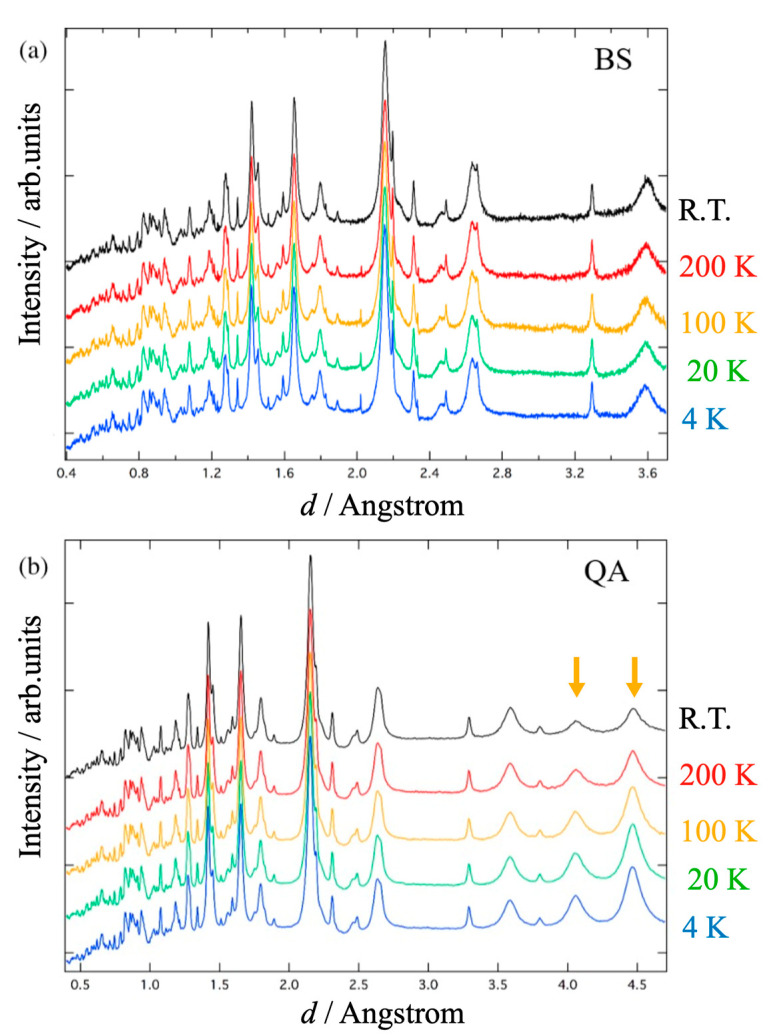
Measured neutron powder diffraction pattern of (Fe_2_O_3_)_0.5_(Al_2_O_3_)_0.5_ solid solution collected at various temperatures by (**a**) BS bank and (**b**) QA bank. 101 and 003 magnetic reflections are depicted by arrows.

Figure 1a shows the temperature dependence of the neutron diffraction patterns of (Fe_2_O_3_)_0.5_(Al_2_O_3_)_0.5_ detected at the BS bank. As depicted in Figure 1a, only slight shifts in peak due to the thermal expansion with no observable structural phase transition were observed from room temperature down to 4 K. While our previous calorimetric investigation [15] suggested anomalous behavior below 25 K, apparent structural change was not detected in the present neutron diffraction experiment.

Figure 1b shows the neutron diffraction patterns of (Fe_2_O_3_)_0.5_(Al_2_O_3_)_0.5_ collected at the QA bank. Relatively large peaks around *d* ~ 4.1 Å and 4.5 Å attributed to the 101 and 003 magnetic reflections, respectively, are observed. Since these reflections have been reported for hematite [22], we suggest that the spin arrangement of the iron sublattices in the solid solution is related to that of pure α-Fe_2_O_3_. In addition, the intensities of the 101 and 003 magnetic reflections seem to decrease gradually with the increase in temperature, while other peaks do not.

**Figure 2:**
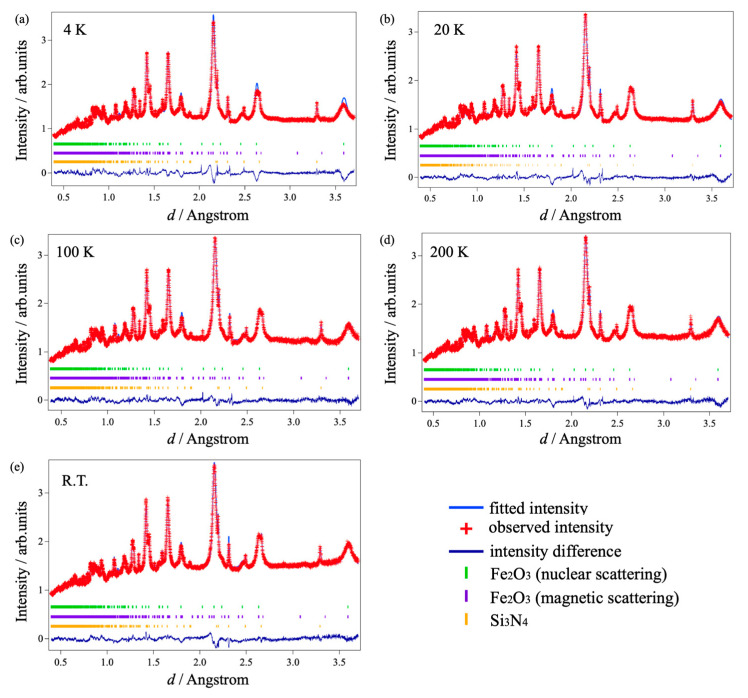
Refined neutron diffraction patterns of (Fe_2_O_3_)_0.5_(Al_2_O_3_)_0.5_ solid solution collected at BS bank. (**a**) 4 K, (**b**) 20 K, (**c**) 100 K, (**d**) 200 K, and (**e**) room temperature.

**Figure 3:**
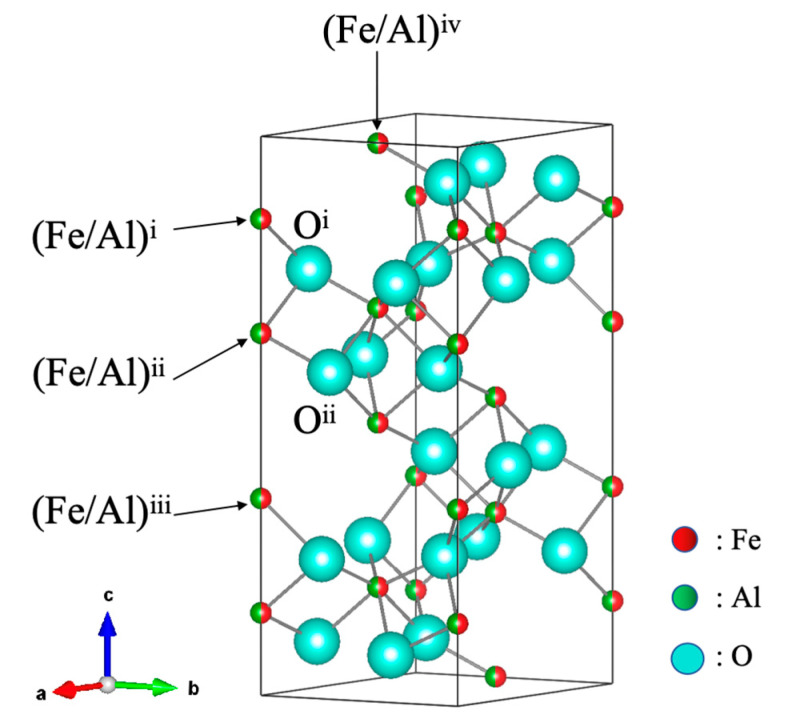
Lattice structure model of (Fe_2_O_3_)_1−x_(Al_2_O_3_)_x_ solid solution drawn by VESTA [23]. The superscript i–iv indicate the equivalent sites obtained by symmetrical operations using single 12*c* and 18*e* positions.

Figure 2a–e represent the Rietveld refined neutron diffraction patterns of (Fe_2_O_3_)_0.5_(Al_2_O_3_)_0.5_ collected at 4 K, 20 K, 100 K, 200 K, and room temperature by the BS bank. A small number of diffraction peaks for silicon nitride (Si_3_N_4_) contamination of the milling vessel were also included in the analysis as the second phase. To evaluate the possibility of remaining starting materials, the Rietveld refinement was examined, assuming the coexistence of γ-Fe_2_O_3_ and γ-Al_2_O_3_. However, the calculated mass fractions of the γ-phases were less than 0.1%, so the analysis was conducted assuming a corundum-type solid solution with a minimal amount of Si_3_N_4_. Although the peak profiles were broadened due to the milled sample, and lower *d*-spacing peaks overlapped considerably, profile fitting could be performed with rather small *R*_wp_. From the lattice and magnetic reflections, the propagation vector was obtained as *k* = (0, 0, 0), indicating that the periodicity of the magnetic structure aligns with the lattice structure without any additional modulation. Through the analysis of the magnetic diffraction peaks detected in the QA bank, the magnetic moments |*M*| were obtained as 1.293 *μ*_B_ and 1.189 *μ*_B_ at 4 K and room temperature, respectively. This slight improvement in |*M*| with the decrease in temperature might be due to the restricted thermal fluctuations of magnetic spins at low temperatures, although the contribution is much smaller in comparison with the effect of aluminum substitution as described later. Table 1 summarizes the structural parameters of (Fe_2_O_3_)_0.5_(Al_2_O_3_)_0.5_ at various temperatures obtained from the Rietveld refinement. Some selected interatomic distances are also listed in Table 2, for which the atomic labels are available in the structural model in Figure 3.

**Figure 4:**
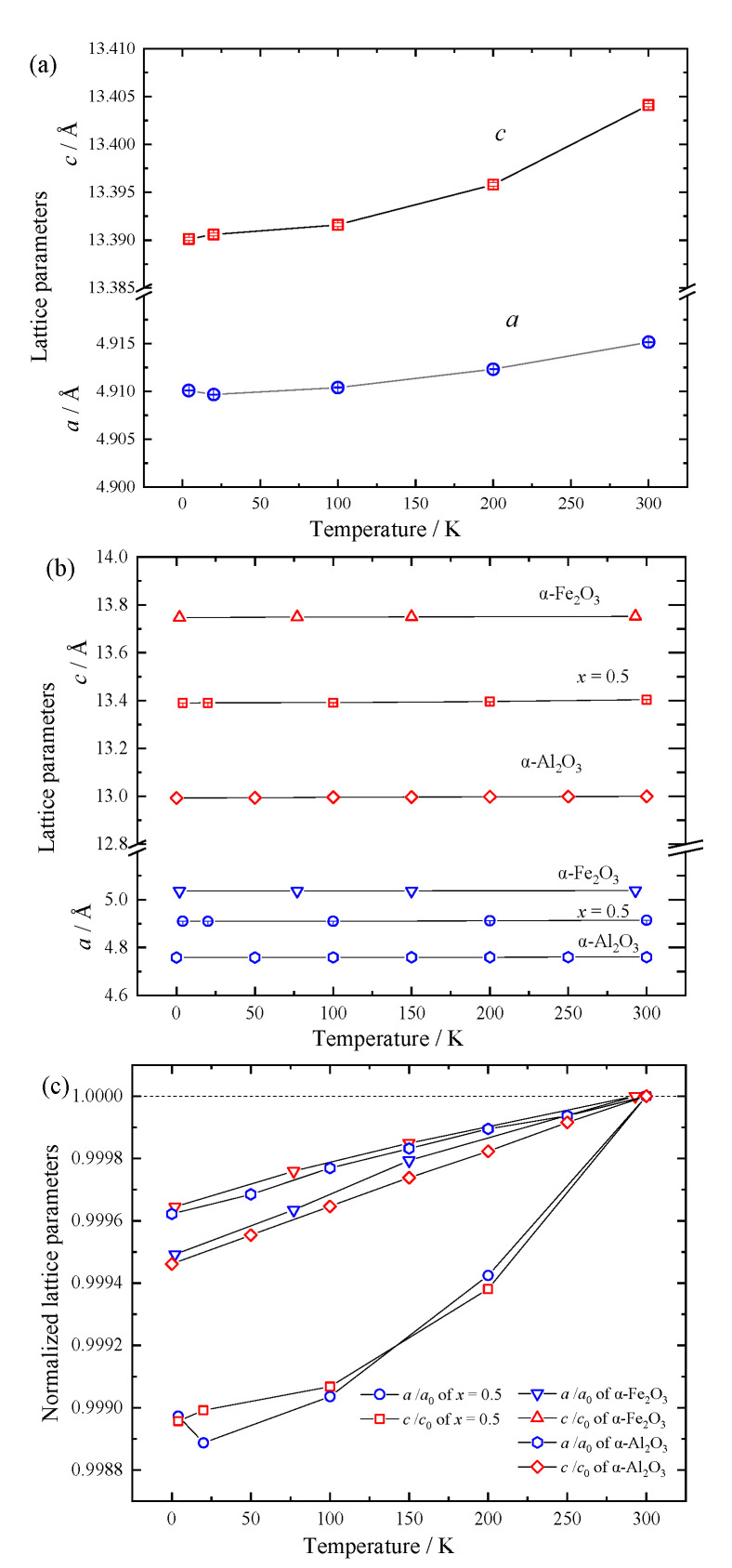
Temperature dependence of the tetragonal lattice parameters of (**a**) (Fe_2_O_3_)_0.5_(Al_2_O_3_)_0.5_, (**b**) the comparative plot of lattice parameters with referenced α-Fe_2_O_3_ [24] and α-Al_2_O_3_ [25,26], and (**c**) normalized parameters based on the room-temperature data (*a*_0_ and *c*_0_).

Figure 4a shows the temperature dependence of the lattice parameters of (Fe_2_O_3_)_0.5_(Al_2_O_3_)_0.5_. Both *a* and *c* increase with temperature. Although the lattice parameters of α-Fe_2_O_3_ and α-Al_2_O_3_ are simultaneously plotted in Figure 4b, the compositional dependence is too significant for direct comparison. To compare the thermal evolutions, the lattice parameters were normalized by each room temperature value and plotted in Figure 4c. Assuming the linear thermal expansion coefficient,

$$\alpha =\frac{1}{l_{0}}\cdot \frac{\Delta l}{\Delta T}$$

the α_a_ and α_c_ of (Fe_2_O_3_)_0.5_(Al_2_O_3_)_0.5_ were evaluated, where *l*_0_ is the lattice parameter at the lower temperature side, and Δ*l*/Δ*T* represents the average temperature gradient of the lattice parameters for the given temperature range. Table 3 shows the thermal expansion coefficients of (Fe_2_O_3_)_0.5_(Al_2_O_3_)_0.5_ compared with α-Fe_2_O_3_ and α-Al_2_O_3_. As expected from Figure 4c, α obtained in the temperature range 4–100 K is lower than that in 200–300 K. The difference in the trend among normalized lattice parameters for (Fe_2_O_3_)_0.5_(Al_2_O_3_)_0.5_, α-Fe_2_O_3_, and α-Al_2_O_3_ in Figure 4c is possibly due to lattice distortion induced during the mechanical alloying process or the cation mixing effect.

The previous calorimetric investigation revealed the higher heat capacity for mechanically alloyed (Fe_2_O_3_)_0.5_(Al_2_O_3_)_0.5_ in comparison with the averaged one of pure α-Fe_2_O_3_ and α-Al_2_O_3_, indicating the lower Debye temperature Θ_D_ [15]. Ruffa [27] suggested the relationship between α and Θ_D_ as $\alpha \left(T\right)=\frac{3k}{2aDr_{D}}{\left(\frac{T}{\Theta_{D}}\right)}^{3}g(x_{D})$ for the cubic lattice assuming Morse potential, where k is the Boltzmann constant, and a, $r_{D}$, and D are the lattice parameters, nearest-neighbor distance, and dissociation energy of morse potential, respectively. $x_{D}$ means $\Theta_{D}\;/T$, and $g\left(x_{D}\right)$ is described as $g\left(x_{D}\right)=\int_{0}^{x_{D}}\frac{x^{4}e^{x}dx}{{\left(e^{x}-1\right)}^{2}}$. Since the cubed term ${\left(\frac{T}{\Theta_{D}}\right)}^{3}$ is strongly attributed to α, i.e., a smaller Θ_D_ results in a larger α, the previous heat capacity result is qualitatively consistent with the larger thermal expansion coefficient.

### 3.2. Compositional Dependence of the Structure of (Fe_2_O_3_)_1−x_(Al_2_O_3_)_x_

**Figure 5:**
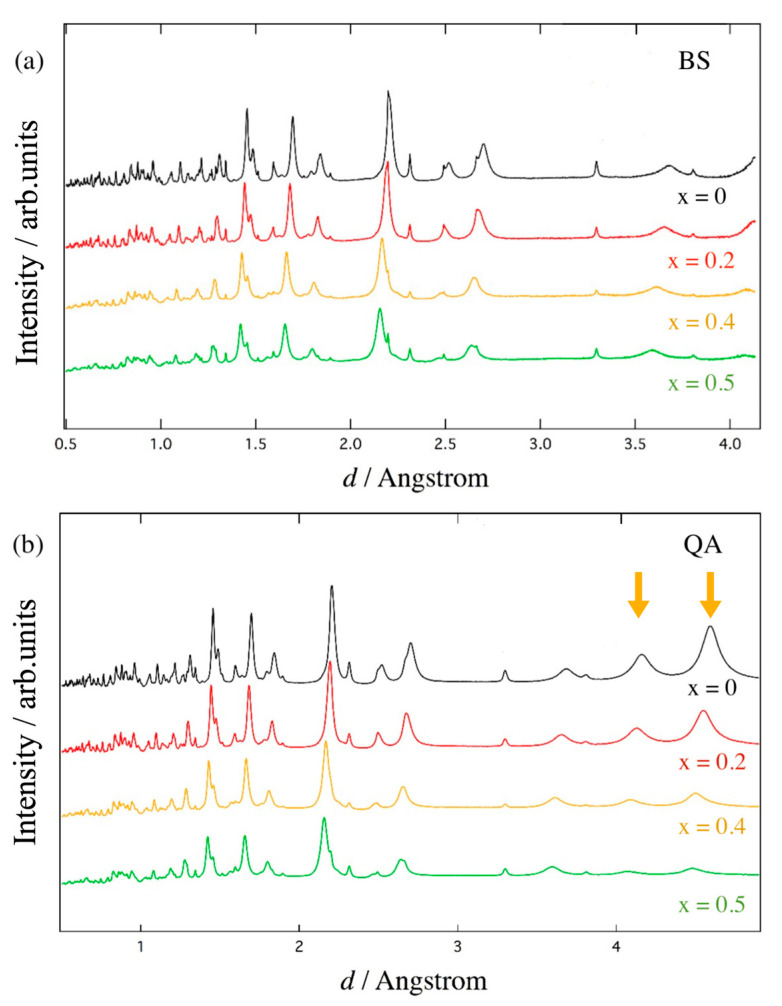
Neutron powder diffraction pattern of (Fe_2_O_3_)_1−x_(Al_2_O_3_)_x_ (x = 0, 0.2, 0.4, and 0.5) at room temperature collected by (**a**) BS bank and (**b**) QA bank. 101 and 003 magnetic reflections are depicted by arrows.

Figure 5 shows the neutron diffraction patterns of (Fe_2_O_3_)_1−x_(Al_2_O_3_)_x_ (x = 0 − 0.5) collected at room temperature through the (a) BS and (b) QA banks. From the diffraction patterns of the BS bank, the overall peaks shift toward lower d-spacings as the x-value increases, indicating a reduction in lattice constants. For the QA bank, the intensity of magnetic reflections around d ~ 4.1 Å and 4.5 Å decreases significantly with the increase in aluminum substitution. This should be due to the dilution of magnetic iron ions by non-magnetic aluminum ions, which weakens the spin–spin interactions. Through the Rietveld refinement, lattice structure information for (Fe_2_O_3_)_1−x_(Al_2_O_3_)_x_ (x = 0, 0.2, 0.4, and 0.5) was obtained and is listed in Table 4. Some selected Fe-O and Fe-Fe bond lengths are shown in Table 5, indicating that increasing aluminum substitution results in a reduction in Fe-O and Fe-Fe bond lengths.

**Figure 6:**
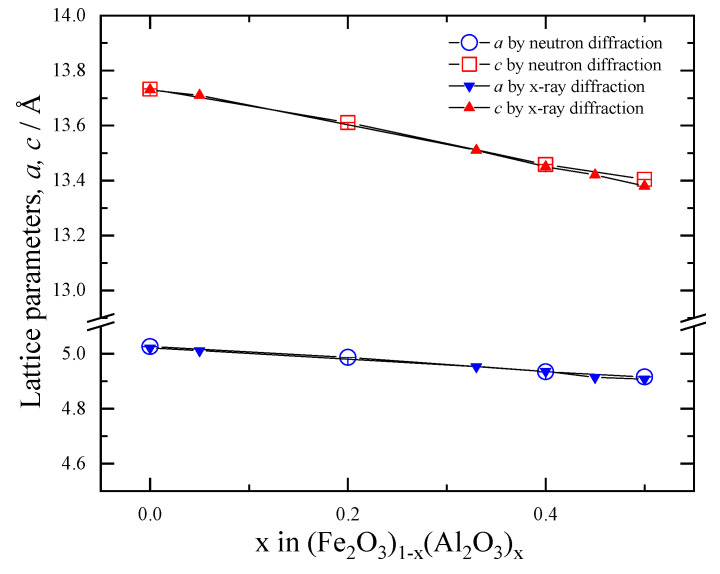
Compositional dependence of lattice parameters of (Fe_2_O_3_)_1−x_(Al_2_O_3_)_x_ (x = 0, 0.2, 0.4, and 0.5) at room temperature. Open symbols present neutron diffraction data, and closed symbols denote previous X-ray diffraction data [12].

Figure 6 plots the compositional dependences of the lattice parameters for (Fe_2_O_3_)_1−x_(Al_2_O_3_)_x_, showing that both lattice parameters *a* and *c* decrease linearly with the aluminum concentration. These lattice parameters agree well with the previous X-ray diffraction results [12], which are depicted by closed symbols. This behavior should be ascribed to the smaller ionic radius of aluminum (0.535 Å) compared to that of iron (0.645 Å) [28,29]. Hence, we suggest that replacing iron ions with smaller aluminum ions reduces the distance between oxygen ions, leading to lattice contraction. Since the Fe-O or Fe-Fe distances vary with the concentration of aluminum, the excellent cycle performance for 0.33 ≤ x ≤ 0.45 [12] is not due to the structural characteristics but the electrochemical properties such as conductivity or equilibrium potential.

**Figure 7:**
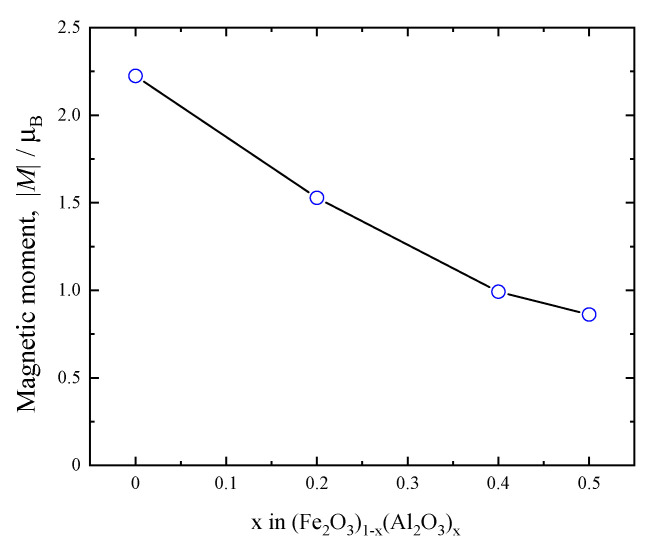
Magnetic moment obtained through the Rietveld refinement using the data from the QA bank of compositional dependence for (Fe_2_O_3_)_1−x_(Al_2_O_3_)_x_ at room temperature.

Figure 7 shows the compositional variation of the magnetic moment |*M*| of (Fe_2_O_3_)_1−x_(Al_2_O_3_)_x_ calculated with the data at the QA bank. For the sample of x = 0 obtained by milling γ-Fe_2_O_3_, the magnetic moment |*M*| is deduced to be 2.224 *μ*_B_, whereas that of α-Fe_2_O_3_ at 300 K is 4.118(6) *μ*_B_ in the MAGNDATA database [30]. This discrepancy might be due to the smaller particle size and possibly the introduced micro-strain as well as poorer crystallinity caused by the mechanical alloying process. Nanoparticle-sized materials often exhibit characteristic behaviors [31,32]. A higher surface ratio with lower coordination numbers results in the reduction in surface exchange interactions and the magnetic moment [33,34]. As the aluminum substitution increases, the magnetic moment continuously decreases. The partial substitution of aluminum with iron reduces the spin–spin interactions, allowing the magnetic state of (Fe_2_O_3_)_1−x_(Al_2_O_3_)_x_ to shift from weak ferromagnetism to a rather paramagnetic state. This behavior is consistent with the previous Mössbauer measurements, showing gradual change in spectra from sextet into doublet profiles [14]. Further comparison with Mössbauer spectroscopy requires simulation, including iron dispersion and spin interaction, which should be conducted in future work.

## 4. Conclusions

The lattice and magnetic structures of mechanically alloyed (Fe_2_O_3_)_1−x_(Al_2_O_3_)_x_ solid solutions were investigated by TOF neutron powder diffraction with temperature and compositional variation. No observable structural phase transition was detected in the neutron diffraction patterns of (Fe_2_O_3_)_0.5_(Al_2_O_3_)_0.5_ from 4 K to room temperature, except for a slight shift due to the thermal expansion. The temperature dependence of the normalized lattice parameters is quite different from that of α-Fe_2_O_3_ and α-Al_2_O_3_, presumably due to lattice distortion or the Fe/Al mixing effect. On the other hand, the lattice parameters of the (Fe_2_O_3_)_1−x_(Al_2_O_3_)_x_ solid solution decrease linearly with the increase in the aluminum concentration at room temperature, as observed in the previous X-ray diffraction experiment. The deduced magnetic moment of (Fe_2_O_3_)_1−x_(Al_2_O_3_)_x_ also decreases with the increase in aluminum substitution due to the reduction in magnetic interaction, as expected from the previous Mössbauer spectroscopy investigation. In addition, the change in the interatomic distances, especially for Fe–Fe, which governs the exchange interactions, should be considered. This investigation would provide essential information on the structure of newly synthesized (Fe_2_O_3_)_1−x_(Al_2_O_3_)_x_ solid solutions.

## Figures and Tables

**Table 1:** Structure parameters of mechanically alloyed (Fe_2_O_3_)_0.5_(Al_2_O_3_)_0.5_ refined from the BS bank data.

Temperature (K)	Atom	Site	*g*	*x*	*y*	*z*	*B*_iso_ (Å^2^)
4	Fe/Al	12*c*	1	0	0	0.14729(2)	0.810(1)
	O	18*e*	1	0.31477(5)	0	0.25	0.939(1)
		*a* = 4.91009(4) Å, *c* = 13.39012(17) Å, *R*_wp_ = 2.4458%					
20	Fe/Al	12*c*	1	0	0	0.14620(2)	0.772(3)
	O	18*e*	1	0.30777(6)	0	0.25	0.763(4)
		*a* = 4.90967(4) Å, *c* = 13.39059(16) Å, *R*_wp_ = 2.3661%					
100	Fe/Al	12*c*	1	0	0	0.14731(3)	0.830(4)
	O	18*e*	1	0.31149(8)	0	0.25	0.832(4)
		*a* = 4.91040(5) Å, *c* = 13.3916(2) Å, *R*_wp_ = 2.5226%					
200	Fe/Al	12*c*	1	0	0	0.14667(2)	0.776(4)
	O	18*e*	1	0.30910(7)	0	0.25	0.743(3)
		*a* = 4.91233(4) Å, *c* = 13.3960(2) Å, *R*_wp_ = 2.0989%					
R.T.	Fe/Al	12*c*	1	0	0	0.14738(2)	0.858(4)
	O	18*e*	1	0.30858(6)	0	0.25	0.745(3)
		*a* = 4.91514(3) Å, *c* = 13.4041(2) Å, *R*_wp_ = 2.4458%					

Space group: $R\bar{3}c$ (hexagonal lattice); *g*: occupancy; *x*, *y*, *z*: fractional coordinates; *B*_iso_: atomic displacement factor.

**Table 2:** Temperature dependences of the selected Fe-O and Fe-Fe distances in (Fe_2_O_3_)_0.5_(Al_2_O_3_)_0.5_ evaluated from Table 1, and superscripts i–iv indicate the corresponding ions in Figure 3.

Temperature (K)	(Fe/Al)^i^–O^i^ (Å)	(Fe/Al)^ii^–O^ii^ (Å)	(Fe/Al)^i^–(Fe/Al)^ii^ (Å)	(Fe/Al)^ii^–(Fe/Al)^iii^ (Å)	(Fe/Al)^i^–(Fe/Al)^iv^ (Å)
4	2.0689(3)	1.8894(2)	2.7506(5)	3.9445(5)	3.3121(3)
20	2.0531(3)	1.8995(2)	2.7800(5)	3.9153(5)	3.2969(3)
100	2.0568(4)	1.8974(3)	2.7504(9)	3.9454(9)	3.3126(5)
200	2.0533(3)	1.9009(2)	2.7687(6)	3.9293(6)	3.3049(3)
R.T.	2.0476(3)	1.9069(2)	2.7511(6)	3.9510(6)	3.3168(3)

**Table 3:** The calculated thermal expansion coefficients of (Fe_2_O_3_)_0.5_(Al_2_O_3_)_0.5_.

	Temperature (K)	α*_a_* (10^−6^ K^−1^)	α*_c_* (10^−6^ K^−1^)
(Fe_2_O_3_)_0.5_(Al_2_O_3_)_0.5_	4–100	0.66(2)	1.15(3)
	200–300	5.76(2)	6.19(5)
α-Fe_2_O_3_ [24]	2–77	1.90	1.53
	150–293	1.44	1.05
α-Al_2_O_3_ [25,26]	2–100	1.50	1.89
	200–300	1.05	1.77

**Table 4:** Structure parameters of (Fe_2_O_3_)_1–x_(Al_2_O_3_)_x_ (x = 0, 0.2, 0.4 and 0.5) at room temperature.

Composition	Atom	Site	*g*	*x*	*y*	*z*	*B*_iso_ (Å^2^)
*x* = 0	Fe/Al	12*c*	1	0	0	0.145556(8)	0.937(1)
	O	18*e*	1	0.30992(4)	0	0.25	0.610(2)
		*a* = *b* = 5.025556(17) Å, *c* = 13.73302(9) Å, *R*_wp_ = 3.6910%					
*x* = 0.2	Fe/Al	12*c*	1	0	0	0.14723(1)	0.870(1)
	O	18*e*	1	0.30862(4)	0	0.25	0.636(2)
		*a* = *b* = 4.98646(2) Å, *c* = 13.61093(10) Å, *R*_wp_ = 2.6605%					
*x* = 0.4	Fe/Al	12*c*	1	0	0	0.146591(13)	0.915(2)
	O	18*e*	1	0.31000(5)	0	0.25	0.774(3)
		*a* = *b* = 4.93405(3) Å, *c* = 13.45815(14) Å, *R*_wp_ = 2.0553%					
*x* = 0.5	Fe/Al	12*c*	1	0	0	0.14738(2)	0.858(4)
	O	18*e*	1	0.30858(6)	0	0.25	0.745(3)
		*a* = *b* = 4.91514(3) Å, *c* = 13.4041(2) Å, *R*_wp_ = 2.4458%					

Space group: $R\bar{3}c$ (hexagonal lattice); *g*: occupancy; *x*, *y*, *z*: fractional coordinates; *B*_iso_: atomic displacement factor.

**Table 5:** Compositional dependences of the Fe-O distances and Fe-Fe distances in the (Fe_2_O_3_)_1–x_(Al_2_O_3_)_x_ at room temperature evaluated using Table 4, and superscripts i–iv indicate the corresponding ions in Figure 3.

*x-*Value	(Fe/Al)^i^–O^i^ (Å)	(Fe/Al)^ii^–O^ii^ (Å)	(Fe/Al)^i^–(Fe/Al)^ii^ (Å)	(Fe/Al)^ii^–(Fe/Al)^iii^ (Å)	(Fe/Al)^i^–(Fe/Al)^iv^ (Å)
0	2.1174(2)	1.9358(1)	2.8687(3)	3.9978(3)	3.3674(1)
0.2	2.0942(2)	1.9240(1)	2.8163(3)	3.9891(3)	3.3539(1)
0.4	2.0679(3)	1.9059(2)	2.7834(4)	3.9457(4)	3.3187(2)
0.5	2.0476(3)	1.9069(2)	2.7511(6)	3.9510(6)	3.3168(2)

## Data Availability

The original contributions presented in this study are included in the article. Further inquiries can be directed to the corresponding author.
